# Iron-sensitive RNA regulation by poly C-binding proteins

**DOI:** 10.1093/nar/gkaf942

**Published:** 2025-09-30

**Authors:** Grant A Goda, Kwame Forbes, Michael E Sullivan, Grace A Eramo, Conner Breen, Douglas F Porter, Paul A Khavari, Daniel Dominguez, Maria M Aleman

**Affiliations:** Department of Chemistry, University of North Carolina, Chapel Hill, NC 27599, United States; Department of Pharmacology, University of North Carolina, Chapel Hill, NC 27599, United States; Department of Pharmacology, University of North Carolina, Chapel Hill, NC 27599, United States; Bioinformatics & Computational Biology, University of North Carolina, Chapel Hill, NC 27599, United States; Department of Pharmacology, University of North Carolina, Chapel Hill, NC 27599, United States; Department of Pharmacology, University of North Carolina, Chapel Hill, NC 27599, United States; Department of Pharmacology, University of North Carolina, Chapel Hill, NC 27599, United States; Program in Epithelial Biology, Stanford University, Stanford, CA 94305, United States; Program in Epithelial Biology, Stanford University, Stanford, CA 94305, United States; Department of Pharmacology, University of North Carolina, Chapel Hill, NC 27599, United States; Bioinformatics & Computational Biology, University of North Carolina, Chapel Hill, NC 27599, United States; Department of Biochemistry & Biophysics, University of North Carolina, Chapel Hill, NC 27599, United States; Lineberger Comprehensive Cancer Center, University of North Carolina, Chapel Hill, NC 27599, United States; RNA Discovery Center, University of North Carolina, Chapel Hill, NC 27599, United States; Department of Pharmacology, University of North Carolina, Chapel Hill, NC 27599, United States; RNA Discovery Center, University of North Carolina, Chapel Hill, NC 27599, United States; Blood Research Center, University of North Carolina, Chapel Hill, NC 27599, United States; McAllister Heart Institute, University of North Carolina, Chapel Hill, NC 27599, United States

## Abstract

Iron is essential for normal cellular function. Homeostatic responses to low iron availability have long been known to rely on posttranscriptional mechanisms. Poly C-binding proteins (PCBPs) are essential RNA-binding proteins that regulate alternative splicing (AS), translation, and RNA stability. They also serve as critical iron chaperones that manage intracellular iron flux. However, the impact of cellular iron levels on the PCBP-directed transcriptome has not been globally evaluated. We found broad transcriptome changes, including AS, in response to low iron availability consistent with numerous operant posttranscriptional mechanisms that sense iron. By comparing AS directed by PCBP1 and PCBP2 to the iron-sensitive transcriptome, we found genes with iron-sensitive PCBP-mediated splicing regulation. We also found that iron chelation-induced splicing changes were attenuated with knockdown of PCBPs. Further, we demonstrate that iron chelation or mutation of PCBP1 iron binding residues enhances PCBP1 RNA association. This work highlights widespread iron-sensitive RNA regulation and identifies PCBP1 and PCBP2 as critical splicing factors contributing to this response.

## Graphical Abstract

**Graphical Abstract:**
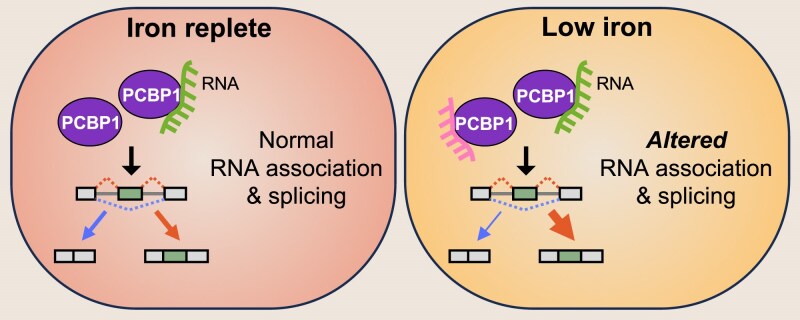

## Introduction

Gene expression and metabolism are intertwined processes that both rely on the availability of metal ions such as iron. Iron supports reactions needed to carry out important cellular functions such as DNA synthesis and oxidative phosphorylation and is essential for the activity of iron-dependent enzymes (e.g. demethylases or phosphatases) [1]. Too much or too little iron is problematic for cell function and can lead to toxicity or stress. Thus, organisms have evolved both systemic and cellular mechanisms to control the availability of iron. RNA regulation is a critical component of intracellular iron homeostasis and involves the RNA binding activity of iron regulatory proteins (IRP) 1 and 2. Through specific interactions with structured iron regulatory elements (IREs) in messenger RNA (mRNA) untranslated regions, IRPs can promote or repress the translation of key iron homeostasis genes. Mechanisms controlling the ability of IRPs to bind RNA are iron-dependent. Thus, when intracellular iron levels are low, IRP–IRE interactions are enhanced leading to increased translation of mRNAs like transferrin receptor 1 (*TFRC*) or decreased translation, like ferritin heavy chain (*FTH1*) [2]. The net effect of this posttranscriptional regulation is to maintain iron levels and enable normal cellular function.

Recently, two additional RNA-binding proteins (RBPs) have been demonstrated as ‘iron-sensitive’. The first, tristetraprolin (also known as ZFP36) is upregulated transcriptionally during iron deficiency to specifically reduce the stability of mRNAs for iron-dependent proteins in the electron transport chain, consequently driving a shift from oxidative phosphorylation to glycolysis to conserve iron [3, 4]. The second RBP with iron-sensitive activity is SRSF7—a splicing factor that promotes the exclusion of alternative exons. Iron disrupts the RNA binding of SRSF7 to target exons, including cell death receptor Fas/CD95, leading to differential regulation of alternative splicing (AS) [5]. These recent discoveries suggest a broader posttranscriptional response to iron levels than previously appreciated and that genes targeted for iron-sensitive regulation extend beyond iron homeostasis genes.

Beyond post-transcriptional control of genes involved in iron import, export, and storage, iron itself is carefully managed in the cell. Protein iron chaperones are used across life forms to metalate specific ferrous iron-dependent proteins or to deliver iron where it is needed intracellularly [6]. Intriguingly, the first mammalian iron chaperone discovered is best known for its well established and canonical role as an RBP. Via a yeast-based screen with a human liver complementary DNA (cDNA) library, poly C-binding protein 1 (PCBP1) was discovered to deliver iron to ferritin, the iron storage complex [7]. Likewise, other PCBP family members (PCBP2–4) share iron chaperone activity [8]. The iron chaperone activity of PCBPs is important for managing iron uptake, iron storage, iron-sulfur cluster formation, heme biosynthesis, and the response to hypoxia [7–15].

Yet, PCBPs are also important regulators of RNA processing, with roles in AS, RNA stability, and translation [16–19]. PCBPs are broadly expressed, especially PCBP1 and PCBP2, and at relatively high concentrations [20]. Seminal studies have shown that these PCBPs bind thousands of cellular mRNAs [in enhanced crosslinking and immunoprecipitation (eCLIP) experiments] and control the RNA processing fate of many genes [21], indicative of a broad role in gene regulation. Indeed, PCBP1 and PCBP2 are both essential for life [18].

The intriguing combination of iron and RNA binding by PCBPs raises interesting questions on how these actions may impact overall PCBP function. For example, does iron binding alter RNA binding? Could PCBPs operate similarly to IRPs? While recent work suggests iron binding and RNA binding by PCBPs are both ‘separable and independent’ [22], two prior *in vitro* studies found that iron reduces PCBP RNA binding [23, 24]. Whether these results translate to cellular RNA binding is not yet clear. Given the broad role of RBPs in modulating gene expression, the potential impact of iron-sensitive RNA regulation by PCBPs warrants further investigation.

Here, we use transcriptomic and in-cell RNA binding approaches to determine how cellular iron levels impact RNA regulation and identify massive scale expression and mRNA splicing changes in response to iron chelation. We investigate the involvement of PCBPs in mRNA regulatory responses to iron chelation and a role for PCBPs in iron-sensitive splicing. We further demonstrate PCBP1–RNA interactions in cells are enhanced by iron chelation, suggesting that iron has a direct impact on the way PCBP1 interacts with mRNA. With PCBPs functioning as both iron chaperones and RBPs, this family of proteins is well situated to integrate iron sensing with control of the cellular adaptive response to low iron via changes in mRNA regulation.

## Materials and methods

### Cell culture and iron chelation treatments

K562 cells, including stable cell lines described below, were cultured in RPMI 1640 (Gibco, #11875-093) with 10% fetal bovine serum (FBS; Corning, #35-015-CV) and 1× Anti-Anti (Gibco, #15240062) and incubated at 37°C with 5% CO_2_. Cultures were periodically tested for mycoplasma contamination with MycoStrip tests (Invivogen, #rep-mysnc-50). For iron chelation experiments, cells were treated with 10 μM 21H7 or 100% dimethyl sulfoxide (DMSO; volume-matched) in RPMI 1640 complete media overnight (∼16 h). K562 cells used in easyCLIP experiments (described below) were treated overnight with 100 μM deferoxamine mesylate (DFO; Sigma, #252750) and 100 μM bathophenanthroline disulfonic acid (BPS; Sigma, #146617) in RPMI 160 complete media. HEK293T cells were cultured in DMEM (Gibco, #11995065) with 10% FBS and 1× Anti-Anti and incubated at 37°C with 5% CO_2_.

### Lentiviral transduction for gene knockdown

For knockdown (KD) of PCBP1, lentiviral particles containing pLKO.1 plasmids with short hairpin RNA (shRNA) genes for Luciferase (shCtrl) or PCBP1 (shPCBP1, see Table 1) and *PurR* for puromycin resistance were generated as described from HEK293T cells [25, 26]. K562 cells were treated with media containing lentiviral particles and 8 μg/ml polybrene, overnight at 37°C with 5% CO_2_. The following morning, cells were spun down (500 × *g*, 3 min) and resuspended in selection media (3 μg/ml puromycin, 10% FBS, RPMI 1640) for 4 days before collection. For dual KD of PCBP1 and PCBP2, we generated lentiviral particles containing pLKO.1 plasmids with shRNA genes as follows: Hygro-shCtrl (scrambled shPCBP1 sequence) and Hygro-shPCBP1 with *HygR* and Puro-shCtrl (luciferase) and Puro-shPCBP2 with *PurR* (see Table 1). K562 cells were treated with media containing lentiviral particles and 8 μg/ml polybrene overnight at 37°C and 5% CO_2_. After 2 days, cells were spun down (500 × *g*, 3 min) and resuspended in selection media (3 μg/ml puromycin, 100 μg/ml hygromycin, 10% FBS, RPMI 1640). Two days later, transduced cells were treated with DMSO or 10 μM 21H7 overnight and collected the following morning.

**Table 1.** Key oligo/plasmid reagents

Reagent	Name	Source	Sequence/identifier	Use
Plasmid	pLKO.1-shPCBP1 (puro)	Brent Graveley, UConn	TRCN0000074668, CCCATGATCCAACTGTGTAAT	Stable KD of PCBP1
Plasmid	pLKO.1-shCTRL (puro)	Brent Graveley, UConn	TRCN0000072243, CTTCGAAATGTCCGTTCGGTT	Stable KD control
Plasmid	pLKO.1-shPCBP1 (hygro)	Dominguez Lab	CCCATGATCCAACTGTGTAATCTCGAGATTACACAGTTGGATCATGGG	Stable KD of PCBP1
Plasmid	pLKO.1-shCTRL (hygro)	Dominguez Lab	Scrambled shPCBP1 sequence, GCATTCTACTACGGAACACTTCTCGAGAAGTGTTCCGTAGTAGAATGC	Stable KD control
Plasmid	pLKO.1-shPCBP2 (puro)	Brent Graveley, UConn	TRCN0000074684, CCCACTAATGCCATCTTCAAA	Stable KD of PCBP2
Guide RNA	AAVS1 single-guide RNA(sgRNA)	Synthego, #10004341-1	UAGUGGCCCCACUGUGGGGU	Used to make K562^flox^ cells
Plasmid	Lox2AAV1	Gift from J. Matthew Taliaferro, U of CO	Donor plasmid with homology to AAV1 safe harbor site [42]	Used to make K562^flox^ cells
Plasmid	pRD-Cre	Gift from Eugene Makeyev, King’s College London	pEM784 from [27]	Stable cell line generation with inducible expression of desired genes
Plasmid	pRD-RIPE	Gift from Eugene Makeyev, King’s College London	pEM791 from [27]	Stable cell line generation with inducible expression of desired genes
Plasmid	pGG732	Dominguez Lab	Flag-tagged WT PCBP1	RMCE in K562^flox^ cells
Plasmid	pGE008	Aleman Lab	Flag-tagged ΔRNA PCBP1	RMCE in K562^flox^ cells
Plasmid	pGE009	Aleman Lab	Flag-tagged ΔFe PCBP1	RMCE in K562^flox^ cells
Plasmid	pBG054	Dominguez Lab	Flag-tagged GFP	RMCE in K562^flox^ cells
Plasmid	pMA076	Aleman Lab	*TMEM222* splicing reporter	Minigene reporter
Oligo	DIS3_F	Integrated DNA Technologies (IDT)	GGACCTGGGCTTCTGTGG	Splicing validation
Oligo	DIS3_R	IDT	AACTTGCAGCTGGTTGTCTG	Splicing validation
Oligo	NQO2_F	IDT	TGGCTAGCGACATCACTGAT	Splicing validation
Oligo	NQO2_R	IDT	GGCCACAGGAAGTATCGAGA	Splicing validation
Oligo	NUMB_F	IDT	AGTAGAAGGGGAGGCAGAGA	Splicing validation
Oligo	NUMB_R	IDT	GGGAGTACGTCTATGACCGG	Splicing validation
Oligo	GOLGA2_F	IDT	AACCCTGAGACAACCACTTCT	Splicing validation
Oligo	GOLGA2_R	IDT	CTGAGATGCCGCCATGCTA	Splicing validation
Oligo	TMEM222_F	IDT	TTTTCCCCATCATCGGCCA	Splicing validation
Oligo	TMEM222_R	IDT	CCAAAGGCCATGTTGTCCTC	Splicing validation
Oligo	CARM1_F	IDT	GGCCACAACAACCTGATTCC	Splicing validation
Oligo	CARM1_R	IDT	GTAGTGCATGGTGTTGGTCG	Splicing validation
Oligo	Reporter_F	IDT	GAACCCACTGCTTACTGGCT	Splicing validation
Oligo	Reporter_R	IDT	TAGGGCCCTCTAGATGCATG	Splicing validation
RNA oligo pool	siCtrl	Dharmacon	ON-TARGETplus Non-targeting Pool, D-001810-10-05	Control KD in HEK293T cells
RNA oligo pool	siPCBP1	Dharmacon	ON-TARGETplus Human PCBP1 (5093)small interfering RNA(siRNA) – SMARTpool, L-012243–01–0005	PCBP1 KD in HEK293T cells

### Generation of K562^flox^ cells

K562 cells were electroporated using the Neon^TM^ Transfection System (Thermo Scientific, #MPK1025) with AAVS1 guide RNA (Synthego), Cas9-NLS (Synthego), and a donor plasmid (generously provided by Matt Taliaferro, University of Colorado Anschultz) containing a blasticidin-resistance cassette flanked by loxP and lox2272 sites under an EF-1α promotor with homology regions to the AAV1 locus that is compatible with the pRD-RIPE system for recombinase-mediated cassette exchange (RMCE) [27]. Cells were selected with 10 μg/ml blasticidin (Millipore Sigma, #SBR00022) and plated for single cell clonal expansion. The final clone was genotyped using Sanger sequencing.

### Generation of stable cells lines with inducible gene expression in K562^flox^

K562^flox^ cells were electroporated using the Neon^TM^ Transfection System with pRD-Cre and pRD-RIPE plasmids (generously provided by Eugene Makeyev, King’s College London) modified to contain Flag-PCBP1, Flag-PCBP1 ΔFe (D82A, E168A, and E350A as in [22]), Flag-PCBP1 ΔRNA (R40A, R124A, and R306A as in [22]), or Flag-GFP and incubated overnight in RPMI 1640 with 10% FBS in an incubator at 37°C and 5% CO_2_. The next day, puromycin (1 μg/ml, final, Thermo Scientific, #A1113803) was added to culture media for selection. Polyclonal stable cell lines were maintained and expanded under puromycin selection for downstream experiments.

### Western blotting

Normalized cell lysates prepared in 1× radioimmunoprecipitation assay (RIPA) buffer (Sigma–Aldrich, #20-188) with 1× Halt Protease Inhibitor Cocktail (Thermo Fisher, #78420) were submitted to sodium dodecyl sulphate (SDS)–polyacrylamide gel electrophoresis and transferred to nitrocellulose membranes. Membranes were blocked with 5% milk or 5% bovine serum albumin (BSA; Fisher, #BP9706100) for 30 min at room temperature. Primary antibodies were incubated overnight at 4°C then washed repeatedly with 1× TBST buffer (Tris-buffered saline, 0.05% Tween-20, Thermo Fisher, #J77500.K2). Blots were incubated with peroxidase-conjugated secondary antibodies for 30 min to 1 h at room temperature followed by repeated TBST washes. Blots were then incubated with up to 4-fold diluted western ECL substrate (Bio-Rad, #1705061) for up to 5 min. Blot chemiluminescence was imaged with a ChemiDoc imager (Bio-Rad). Band intensities were measured with ImageJ software. Antibodies used are as follows: Rabbit anti-PCBP1 polyclonal (1:2000, MBL International Corp, #RN024P), Rabbit anti-FTH1 polyclonal (1:1000, Cell Signaling, #3998S), Rabbit anti-IRP2 (D6E6W) monoclonal (1:1000, Cell Signaling, #37135S), Rabbit anti-α-Tubulin polyclonal (1:5000, Cell Signaling, #2144S), Rabbit anti-Vinculin polyclonal (1:5000, Proteintech, #26520-1-AP), Mouse anti-Flag antibody, clone M2 (1:2000, Millipore Sigma, #F1804), Goat anti-Rabbit-HRP-conjugated polyclonal (1:10 000, Jackson ImmunoResearch, #111035045), Donkey anti-Rabbit-HRP-conjugated polyclonal (1:10 000, Jackson ImmunoResearch, #711035152). For some blots, total protein was measured immediately after transfer using 5% Ponceau S stain in 5% acetic acid for 1 min followed by rinsing in diH_2_O and imaging with a ChemiDoc imager.

### mRNAseq

RNA isolated from cells after treatments [iron chelation, PCBP1 KD and overexpression (OE)] was sent to Novogene (Sacramento, CA) for polyA selection and library preparation followed by Illumina sequencing (paired end, 150 cycles). On average, 10 GB of data per sample were collected. For RNA isolated from cells after dual KD with and without iron chelation, libraries were prepared with the KAPA Hyperprep mRNA kit (Roche, #08098123702) with NEBNext Multiplex Oligos (NEB, #E7335S, E7500S, E7710S, E7730S). Final libraries were sent to Novogene for Illumina sequencing (paired end, 150 cycles). On average, 11 GB of data per sample were collected.

### mRNAseq analysis

Quality of sequencing fastq files was checked using FastQC [28] and MultiQC [29]. Salmon (v1.10.2) [30] was used for transcript abundance quantification which was then analyzed for differential gene expression using DESeq2 (v3.19) [31]. Significant genes were quantified by passing a read count threshold >10, having an absolute log_2_ fold change >1 by Wald test and an adjusted *P*-value <.05 [by Benjamin–Hochberg (BH) method]. To capture AS, reads were first mapped to human genome build hg38 from Ensembl [32] using STAR (v2.7.11b) [33]. The resulting bam files were then analyzed with rMATS (v4.3.0) [34]. Significant AS events had an false discovery rate (FDR) <0.05, an absolute [Δ percent spliced in (ΔPSI)] >5 percent, and either an average inclusion read count or exclusion read count ≥ 10. Splicing heatmaps were built using R package pheatmap, with cluster_cols and cluster_rows set to TRUE. Gene ontology (GO) and Gene set enrichment analysis (GSEA) were performed using functions from the R/Bioconductor package, clusterProfiler [35], including enrichGO and GSEA, respectively. GSEA of Hallmark [36] gene sets was performed on pre-ranked lists of genes with a ranking metric defined as log_2_(FC) × (1 − *P*-value) and using the msigdbr package in R. GO analysis for significant splicing events used a custom background of transcripts (at the gene level) with detected splicing events. Chi-square tests were used to determine significant differences between observed and expected results. Hypergeometric tests were used to calculate the significance of overlapping datasets, where only co-detected genes or events were used as background. Spearman’s rank correlation test was used for correlation analyses.

### Hypoxia RNAseq

mRNAseq data of K562 cells treated with normoxia (21% oxygen) or hypoxia (1% oxygen) for 3 days by Jain *et al.* [37] (GEO Accession: GSE144527) was analyzed using GEO2R (NCBI) which computes differential gene expression using DESeq2 via Wald test. Significant genes had adjusted *P*-values <.05 and an absolute log_2_ fold change >1.

### Splicing event validation

Significant alternatively spliced exon events having a FDR <0.05, an absolute ΔPSI > 5 percent, and having an average inclusion read count or exclusion read count > 10, were considered candidate spicing events and were further evaluated for: (i) having clear annotation in UCSC Genome Browser [38], (ii) construction of a pair of polymerase chain reaction (PCR) primers in Primer3web [39], and (iii) using the UCSC In-Silico PCR tool, PCR primers produced between two and three output files in fasta format that contain all sequences in the database that lie between and include the primer pair [38].

K562 cells were treated with iron chelation (21H7), PCBP1 KD or PCBP1 OE with five biological replicates, as described above. RNA was isolated from treated K562 cells using phenol:chloroform extraction and cDNA was produced using the High-Capacity cDNA Reverse Transcription Kit (Invitrogen, #4368814) or using 20mer anchored Oligo dT primers (IDT, #51-01-15-08) with Superscript III or Superscript IV [Invitrogen, #18080044, #18090050)]. For dual KD, RNA was isolated with RNeasy Kit (Qiagen, #74104) with DNase I treatment (Qiagen, #79254) and cDNA was prepared as above. Primer oligos were designed targeting flanking exons of the alternative exon and synthesized by IDT. PCRs were performed using Phusion polymerase (NEB, #M0530L), 1× HF Buffer, 200 μM dNTPs, 0.5 μM forward and reverse primers, with or without 3% DMSO, and 40 ng cDNA with 27 or fewer cycles. Some PCR products were run on 6% tris-borate-ethylenediaminetetraacetic acid (EDTA)-Urea gels (Invitrogen, #EC6865BOX) which were then stained with SYBR-Gold DNA stain (Invitrogen, #S11494) for 5 min with shaking before imaging on a ChemiDoc imager. Other PCR products were run on 2 or 3% agarose gels containing Sybr Safe DNA stain (Invitrogen, #S33102) and imaged on a ChemiDoc imager.

Band intensities for PCR products representing included and excluded alternative exons were measured with ImageJ software. For each biological replicate, we calculated the PSI of the included exon product by dividing the band intensity of the included exon by the total included and excluded splicing product intensities. ΔPSI values per biological replicate were calculated by subtracting control conditions from test conditions. Two-tailed *t*-tests were used to compare PSI or ΔPSI means between two conditions. For PCBP1 KD or OE ΔPSI comparisons, we used a one-sample *t*-test versus a hypothetical mean of 0. When testing PSI means across four conditions, we used a one-way analysis of variance (ANOVA) test with Šídák’s multiple comparisons test for adjusted *P*-values.

### Splicing mini-gene reporter

A three-exon mini-gene splicing reporter was constructed by cloning a fragment of the *TMEM222* gene (chr1:27 330 735–27 332 093) into a pcDNA3 backbone under a cytomegalovirus promoter. HEK293T cells were transfected with 100 ng (per 12-well) of reporter plasmid (pMA076). Four hours after transfection, cells were treated with DMSO or 30 μM 21H7 for 4 h, followed by cell collection and RNA isolation using RNeasy Kit (Qiagen, #74104) with DNase I treatment (Qiagen, #79254). cDNA was prepared using 20mer anchored Oligo dT primers (IDT, # 51-01-15-08) with Superscript III [Invitrogen, #18080044)]. Primers specific to the reporter transcript were used to amplify the mini-gene by PCR (see Table 1). PCR products were run on 2% or 3% agarose gels containing Sybr Safe DNA stain (Invitrogen, #S33102) and imaged on a ChemiDoc imager. Analysis of reporter splicing events was performed as described in the section above.

### Ultraviolet crosslinking and immunoprecipitation (easyCLIP)

K562 cells were treated overnight with DFO and BPS as described above (Cell Culture and Iron Chelation Treatments). The easyCLIP protocol was followed as described [40] with the following adjustments. At harvest, K562 cells (∼45 million per condition) were spun at 200 ×*g* for 5 min, media removed by aspiration, then resuspended in 3 ml 4°C phosphate buffered saline (PBS). The PBS/cell mixture was delivered to a 10 cm plate on a bed of ice and ultraviolet (UV; 254 nm) cross-linked at 400 mJ/cm^2^. After lysis, polyclonal rabbit anti-PCBP1 antibodies (MBL International Corp, #RN024P) were used to immunoprecipitate PCBP1. Bound RNA libraries were prepared and sequenced (paired end, 150 cycles) on a MiSeq machine and analyzed according to previous methods [40], mapping to the hg38 genome and counting reads per feature with HTSeq. Summed counts per gene were normalized and log_2_ fold changes calculated with DESeq2.

### Formaldehyde crosslinking and RNA immunoprecipitation (RNA-immunoprecipitation and sequencing)

RIP experiments were performed as described [41] with the following modifications. Flag-PCBP1, Flag- PCBP1 ΔFe, Flag-PCBP1-ΔRNA, or Flag-GFP were expressed in K562^flox^ stable cell lines by treatment with 200, 1000, 200, or 500 ng/ml doxycycline (Dox; Millipore Sigma, #D9891), respectively, overnight. Dox-treated cells were also treated with either DMSO (200 000 cells/ml) or 10 μM 21H7 (400 000 cells/ml) overnight. The next day, cells were counted, washed twice in PBS, and rotated for 30 min at 4°C in 10 ml of 0.3% formaldehyde (Fisher, #28906) in PBS. Crosslinking was quenched with 200 mM glycine (final, Fisher, #BP381) for 5 min, with rotation, at room temperature then washed twice with cold PBS before resuspension at 5 million cells/ml. Cells were pelleted and snap frozen with a dry ice-ethanol bath and stored at −80°C.

Protein A/G PLUS agarose beads (25 μl per cell pellet, Santa Cruz Biotechnology, #sc-2003) were washed three times in blocking buffer (0.5% BSA in 1× PBS), incubated overnight, with rotation, at 4°C temperature with 10 μl M2 anti-Flag antibody (Millipore Sigma, #F1804), and before use were washed three times with fRIP buffer [25 mM Tris-HCl, pH 7.5, 0.5% NP-40 Alternative (Millipore Sigma, #492016), 5 mM EDTA, pH 8.0, 150 mM KCl, 0.5 mM dithiothreitol (DTT), 1× Halt protease inhibitor cocktail, 1:200 SUPERaseIN]. RIPA buffer [50 mM Tris, pH 8.0, 1% Triton X-100, 0.5% sodium deoxycholate, 0.1% SDS, 5 mM, EDTA, pH 8.0, 150 mM KCl, 0.5 mM DTT, 1× Halt protease inhibitor cocktail, 1:200 SUPERaseIN] was added to each cell pellet. Each sample was sonicated twice (30% amplitude, 2 × 30 s on). All samples were centrifuged at 4°C for 15 min at 15 000 × *g* and supernatant was transferred to a new tube. Fifty microliters from each sample was pulled as inputs and stored at −80°C. Samples were normalized by relative expression of Flag-tagged proteins detected by western blot. Cell lysates and beads were incubated overnight at 4°C with rotation. Beads were rinsed once with 1 ml cold fRIP buffer, then washed three times in PolII chip buffer (50 mM Tris–HCl, pH 7.5, 140 mM NaCl, 1 mM EDTA, pH 8.0, 1 mM EGTA, pH 8.0, 1% Triton X-100, 0.1% sodium deoxycholate, 0.1% SDS), twice in high salt PolII chip buffer (50 mM Tris-HCl, pH 7.5, 500 mM NaCl, 1 mM EDTA, pH 8.0, 1 mM EGTA, pH 8.0, 1% Triton X-100, 0.1% sodium deoxycholate, 0.1% SDS), and once in LiCl buffer (20 mM Tris, pH 8.0, 0.5% NP-40 Alternative, 1 mM EDTA, pH 8.0, 250 mM LiCl, 0.5% sodium deoxycholate). Beads were rotated 5 min at 4°C before centrifugation at 1200 × *g* for 2 min at 4°C for washes. Sample tubes were changed after the first and final wash. After washes, beads were resuspended in 56 μl water and 59 μl of reverse crosslinking buffer (5 mM DTT, 1:5 proteinase K, 1:100 SUPERaseIN). Samples were incubated at 42°C for 1 h, 55°C for 1 h, and 65°C for 30 min. RNA was subsequently extracted with trizol/chloroform and cleaned with the Zymo RNA Clean and Concentrator-5 columns (Zymo Research, #R1013) with DNA digestion.

Library preparation of isolated RNA from immunoprecipitations (IP) or from whole cell lysates (inputs) was performed with the KAPA RNA Hyperprep Kit with RiboErase (Roche, #KK8560) using NEBNext Multiplex Oligos (adaptors and single index primers, New England Biolabs, #E7335, #E7500, #E7730) compatible with Illumina sequencing. Pooled libraries were sequenced (paired end, 150 cycles) on an Illumina NovoSeq X Plus by Novogene. Quality of sequencing fastq files was checked using FastQC [28] and MultiQC [29]. Salmon (v1.10.2) [30] was used for transcript abundance quantification which was then analyzed for enrichment of bound RNAs using DESeq2 (v3.19) [31] with a Wald test comparing gene level read counts of immunoprecipitated RNAs to read counts of those RNAs in input samples. Genes with significant positive enrichments (log_2_ fold change > 1, *P*_adj_<.05) for Flag-GFP and Flag-PCBP1 ΔRNA were considered as background and removed from the list of Flag-PCBP1-associated RNAs.

## Results

### Iron chelation broadly alters the transcriptome

**Figure 1.**
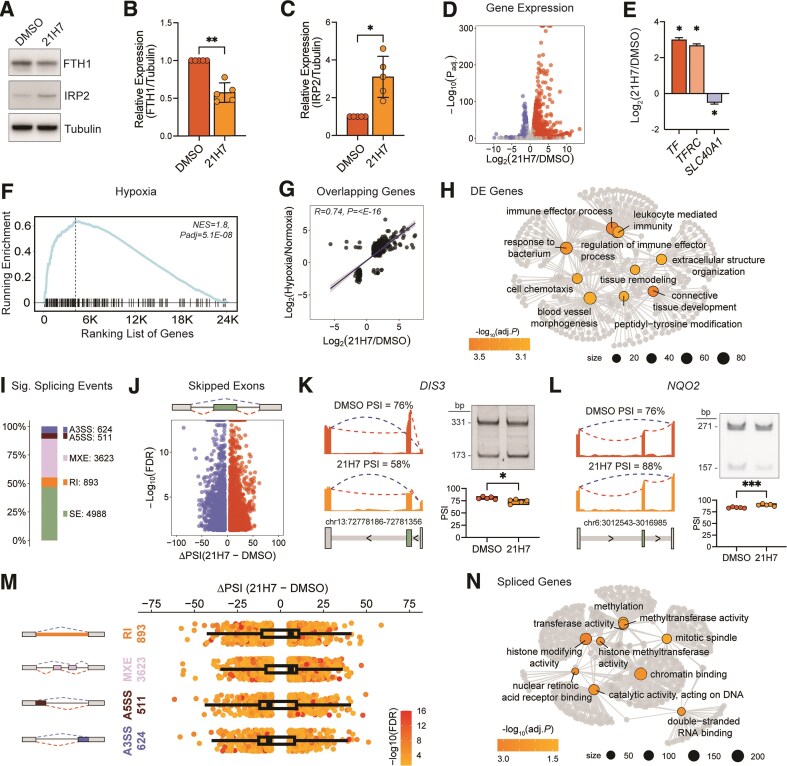
The transcriptome after iron chelation. (**A**) Western blots of FTH1 and IRP2 after treatment of K562 cells with iron chelator, 21H7, or DMSO, *n*= 5. Tubulin was used as loading control. (**B**, **C**) Western blot quantification of FTH1 and IRP2, respectively, after treatment. Data are mean ± standard deviation (SD). *P<.05, **P<.01 by two-tailed, paired *t*-test. (**D**) Volcano plot of differentially expressed genes (DEG), after iron chelation, *n*= 3. Blue points represent downregulated genes, and red points represent upregulated genes. Significance tested by Wald test with BH method for adjusted *P*-values. (**E**) Bar plot of key DEGs (*TF, TFRC, SLC40A1*) after iron chelation. Data are mean ± standard error of the mean (SEM). *P <.05 by Wald test. (**F**) Enrichment of hypoxia genes after iron chelation. Normalized enrichment score (NES) and adjusted *P*-value by GSEA. (**G**) Scatter plot of DEGs that overlap significantly between iron chelation (21H7 versus DMSO) and Hypoxia (1% versus 21% O2 at 3 days) [37] with Spearman’s rank correlation. (**H**) Network plot of GO terms from significantly DEGs after iron chelation. GO term node size and color reflect gene count and significance, respectively (over-representation test with adjusted *P*-value by BH method). Gray dots are individual genes, and gray lines connect each gene to their associated GO term. (**I**) Bar plot of the proportion and count of significant AS events in 21H7 versus DMSO for each event type detected by rMATS. Skipped exons (SE), alternative 5′ splice site (A5SS), alternative 3′ splice site (A3SS), retained intron (RI), mutually exclusive exon (MXE). (**J**) Volcano plot of significant SEs after iron chelation by likelihood ratio test with FDR, by BH method (in rMATS). Blue points are excluded exon events, and red points are included exon events. (**K**, **L**) **Left**, RNAseq coverage tracks of *DIS3* and *NQO2*, respectively, with mean PSI reported. **Right**, Reverse transcriptase-polymerase chain reaction (RT-PCR) analysis and quantification for *DIS3* and *NQO2* mRNA, respectively, with and without iron chelation. *P<.05, ***P<.001 by two-tailed *t*-test. (**M**) Scatter and overlaid box plot of significant AS events after iron chelation. Each dot is an event colored by degree of significance (likelihood ratio test with FDR by BH method). Box plot shows median of AS event PSI. (**N**) Network plot of GO terms from significant AS genes after iron chelation. GO term node size and color reflect gene count and significance, respectively (over-representation test with adjusted *P*-value by BH method). Gray dots are individual genes, and gray lines connect each gene to their associated GO term.

Our overall hypothesis is that PCBPs regulate RNA in an iron-sensitive manner. To explore this, we first evaluated how the transcriptome changed in response to iron chelation. We performed deep mRNAseq on RNA from K562 cells treated overnight with or without a cell-permeable iron chelator (21H7 [43], *n* = 3). K562 cells, an erythroid leukemia cell line, were chosen for these experiments due to their known PCBP-directed RNA regulatory activity [44–47], as well as the public availability of PCBP KD data and eCLIP data from the ENCODE consortium [48] that identifies K562 PCBP target RNAs. We opted for overnight treatment to represent a steady state response to low iron availability. To confirm that the 21H7 iron chelator functioned as expected, we validated known changes in protein levels for ferritin heavy chain (FTH1) [49] and IRP2 [50] by western blot (*n* = 5). With iron chelation, FTH1 was decreased, while IRP2 was increased (Fig. 1A–C). We observed large-scale changes in mRNA levels; 1899 genes were significantly upregulated (*P* <.05 and log_2_ fold change >1), and 586 genes were significantly downregulated (*P* <.05 and log_2_ fold change <−1; Fig. 1D and Supplemental Table S1). We evaluated the mRNA levels of genes known to change in response to low iron in K562 cells. We observed increased mRNA levels for transferrin (*TF*) and transferrin receptor 1 (TFR1, gene *TFRC*) [51] and decreased mRNA levels for ferroportin (*SLC40A1*) [52] (Fig. 1E). Over 500 genes were more upregulated than *TFRC* and over 100 genes were more downregulated than *SLC40A1* which highlights the extent and robustness of gene expression changes in response to low iron availability.

We performed GSEA of ‘hallmark’ gene sets [36] and observed significant enrichment of hypoxia genes (Fig. 1F). Iron chelators, such as desferoxamine (DFO), are often used to simulate hypoxia *in vitro* and *in vivo* [53–55], thus enrichment for this pathway was expected. Indeed, we found a significant positive correlation in differential gene expression between our iron chelation data set and a hypoxia treatment data set [37] in the same cell line (Fig. 1G). With iron chelation, we also found significant enrichment by GSEA for multiple signaling pathways including TNFα signaling (Supplemental Table S2), consistent with prior work showing TNFα signaling promotes sequestration of iron [56, 57]. GO analysis of differential genes identified hits amongst immune response, tyrosine phosphorylation, and lymphoid and myeloid cell differentiation (Fig. 1H and Supplemental Table S3), highlighting the broad role of iron (and regulation of gene expression) in pathways for growth and development. Together, these data clearly show pervasive transcriptome-wide changes after modulating the availability of a single ion in cells and changes in expected and previously underappreciated iron-sensitive pathways.

Because changes in mRNA levels result from multiple layers (e.g. altered transcription or RNA decay), we next evaluated AS in response to iron chelation. We detected thousands of significant AS events, including SE, retained introns, MXEs, and 5′ and 3′ alternative splice sites (Fig. 1I). To the best of our knowledge such a dramatic impact of iron chelation on RNA splicing has not been reported. The most abundant splicing event type was SE, which occur when an alternative exon is included or excluded from a given transcript (Fig. 1J, top). We measured PSI values and found among the nearly 5000 significant SE events, more were included than excluded (2714 included versus 2274, *P*= 4.66e-10 by Chi-squared test, Fig. 1J, bottom). We validated SE events in *DIS3* (Fig. 1K) and *NQO2* (Fig. 1L), representing both decreased and increased exon inclusion, respectively. DIS3 is a component of the RNA exosome with ribonuclease activity involved in cell differentiation pathways [58–60] and NQO2 is an oxidoreductase involved in metabolism [61, 62]. Among the other splicing events, retained introns and MXEs had increased levels of inclusion (*P* = 8.56e-06 and *P* = 1.30e-12, respectively, by Chi-squared test), whereas alternative 5′ and 3′ splice sites both exhibited decreased inclusion (*P*= 2.34e-05 and *P*= 1.26e-07, respectively by Chi-squared test) (Fig. 1M). Genes affected at the level of splicing were involved in pathways such as histone methylation and RNA binding (Fig. 1N and Supplemental Table S4).

Overall, iron chelation had a profound impact on the transcriptome of K562 cells and the abundance of changes both in gene expression and AS support a model where co- or posttranscriptional mRNA regulation is highly sensitive to cellular iron levels.

### PCBP1-directed transcriptome alternations

**Figure 2.**
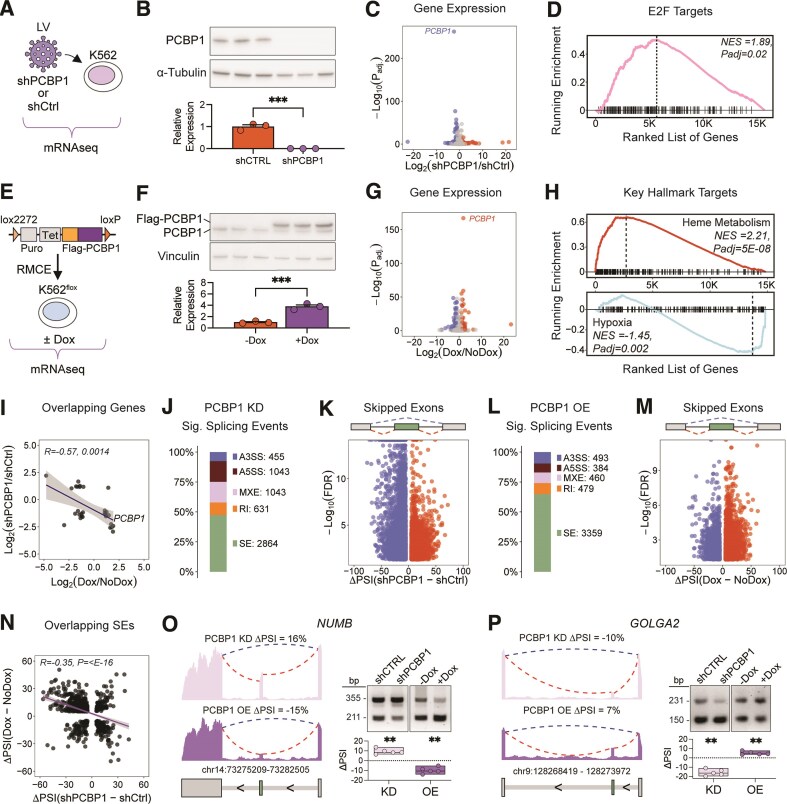
The PCBP1-regulated transcriptome. (**A**) Schematic of the mRNA-seq experiment of K562 cells transduced with shPCBP1 or shCtrl, *n* = 3. (**B**) Western blot and quantification of PCBP1 after KD. Tubulin was used as a loading control. ***P= 0.003 by two-tailed *t*-test. (**C**) Volcano plot of DEGs comparing shPCBP1 versus shCtrl. Blue points represent downregulated genes, and red points represent upregulated genes. Significance tested by Wald test with BH method for adjusted *P*-values. (**D**) Enrichment of hallmark ‘E2F targets’ after PCBP1 KD by GSEA. NES and adjusted *P*-values calculated by GSEA. (**E**) Schematic of the mRNA-seq experiment of K562 cells with Dox-inducible Flag-PCBP1 OE, *n* = 3. (**F**) Western blot and quantification of +Dox and −Dox for Flag-PCBP1 and endogenous PCBP1. Vinculin was used as a loading control. ***P= 0.0009 by two-tailed *t*-test. (**G**) Volcano plot of DEGs comparing +Dox versus −Dox. Blue points represent downregulated DEGs, and red points represent upregulated DEGs. Significance tested by Wald test with BH method for adjusted *P*-values. (**H**) Key gene sets altered after PCBP1 OE. NES and adjusted *P*-values calculated by GSEA. (**I**) Scatter plot of overlapping significant DEGs between PCBP1 KD and PCBP1 OE, with Spearman’s rank correlation. (**J**) Bar plot of the proportion and count of significant AS events in with PCBP1 KD for each event type. (**K**) Volcano plot of significant SEs with PCBP1 KD. Significance by likelihood ratio test with FDR by BH method. (**L**) Bar plot of the proportion and count of significant AS events in PCBP1 OE for each event type. (**M**) Volcano plot of significant SEs for PCBP1 OE. Significance by likelihood ratio test with FDR by BH method. (**N**) Scatter plot of overlapping significant SEs between PCBP1 OE and PCBP1 KD with Spearman’s rank correlation. (**O**) Left, RNAseq coverage tracks of *NUMB* after PCBP1 KD or OE. Mean ΔPSI of each condition reported. **Right**, RT-PCR analysis and dot plot of ΔPSI for *NUMB* after PCBP1 KD or OE; *n* = 5, **P<.01 by one-sample *t*-test versus a hypothetical mean of 0. (**P**) Left, RNAseq coverage tracks of *GOLGA2* after PCBP1 KD or OE. Mean ΔPSI of each condition reported. **Right**, RT-PCR analysis and dot plot of ΔPSI for *GOLGA2* after PCBP1 KD or OE; *n* = 5, **P<.01 by one-sample *t*-test versus a hypothetical mean of 0.

We reasoned we could detect iron-sensitive PCBP RNA regulatory activity by comparing changes in gene expression and AS between iron chelation datasets and PCBP KD or OE datasets. Therefore, we performed deep mRNAseq after PCBP1 shRNA-mediated KD or inducible PCBP1 OE in K562 cells. To KD PCBP1, we treated K562 cells with lentiviral particles containing a shRNA gene targeting PCBP1 (shPCBP1) or luciferase-targeting shRNA (shCtrl) (Fig. 2A, *n* = 3). Western blotting for PCBP1 confirmed near complete depletion after viral transduction and selection (Fig. 2B). We found 296 significantly DEGs with PCBP1 KD, and as expected PCBP1 itself was the most significantly downregulated, further confirming KD (Fig. 2C and Supplemental Table S5). A single ‘hallmark’ gene set was enriched with PCBP1 KD, E2F Targets (Fig. 2D), which includes genes involved in DNA replication and cell cycle control (Supplemental Table S2). This finding is consistent with prior results showing that loss of PCBP1 delays cell cycle progression through the G2/M transition in HEK293 cells [22]. GO terms enriched with PCBP1 KD included extracellular matrix organization and metallopeptidase activity (Supplemental Table S3).

To induce PCBP1 OE in K562 cells, we first generated K562^flox^ cells using Cas9/sgRNAs targeted to the AAV1 locus and a linearized plasmid containing a blasticidin resistance gene flanked by lox2722 and loxP sites [42]. Subsequently, we used RMCE [27] to integrate a cassette containing Flag-tagged PCBP1 under the control of a tet-inducible promoter and puromycin resistance (Fig. 2E). After induction with Dox, PCBP1 was four-fold overexpressed (Fig. 2F, *n* = 3). mRNAseq identified 251 significantly DEGs with PCBP1 OE. As expected, the most significant DEG was PCBP1 itself (Fig. 2G and Supplemental Table S6). GSEA found the iron pathway, heme metabolism, significantly enriched after PCBP1 OE, while hypoxia was significantly downregulated (Fig. 2H and Supplemental Table S2). GO terms enriched with PCBP1 OE included vascular development, cytokine receptor binding, and response to reactive oxygen species, among many others (Supplemental Table S3). These hits with PCBP1 OE show a striking connection between PCBP1, iron, and gene regulation. We observed a significant negative correlation in fold changes between PCBP1 KD and OE (Fig. 2I), underscoring that these are PCBP1-directed changes in mRNA levels.

As a splicing factor, loss of PCBP1 caused many changes in AS, especially SE (Fig. 2J and K and Supplemental Fig. S1A). After PCBP1 KD, changes in SE accounted for >50% of significant alternative events, with more exons skipped than included (4177 skipped and 2218 included, *P*< 2.2e-16 by Chi-squared test, Fig. 2K). Likewise, PCBP1 OE also preferentially impacted SE (Fig. 2L and M and Supplemental Fig. S1B) but unlike PCBP1 KD, we saw more exon inclusion than exclusion with PCBP1 OE (1233 skipped and 2126 included, *P*< 2.2e-16 by Chi-squared test, Fig. 2M). GO analysis for splicing events sensitive to PCBP1 KD involved DNA nuclease activity, phospholipid binding, and cilium assembly, among others (Supplemental Fig. S1C and Supplemental Table S4) Together, these data indicate that PCBP1 primarily promotes exon inclusion, as shown previously [45].

Comparing significant SE events across PCBP1 KD and OE datasets, we found 725 overlapping events, which was found to be significant (*P* < 6e-184 by hypergeometric test). Amongst the overlapping events, about 61% (439) occurred with opposing inclusion/exclusion depending on depletion or over-expression (Fig. 2N), resulting in an overall negative correlation (R = −0.35, *P* < 2.2e-16 by Spearman correlation). We validated SE events in *NUMB* and *GOLGA2* mRNAs (*n* = 5 per group). For *NUMB*, this exon was more included with PCBP1 KD whereas it was more skipped with OE (Fig. 2O). In contrast, this *GOLGA2* was more skipped with PCBP1 KD and more included with PCBP1 OE (Fig. 2P). NUMB is an endocytic adaptor protein and mutations in a NUMB-associated complex member causes iron accumulation and neurodegeneration in humans [63, 64]. GOLGA2, also known as GM130, is a structural protein involved in the Golgi apparatus and chromosome segregation [65, 66]. The 439 opposing inclusion/exclusion events, likely core PCBP1-regulated splicing targets in K562 cells, are enriched in genes associated with deacetylase activity (Supplemental Fig. S1D). These genes included histone deacetylase and sirtuin family members and both groups have been implicated in control of iron homeostasis [67, 68].

### Overlap between iron-sensitive and PCBP-directed transcriptomes

**Figure 3.**
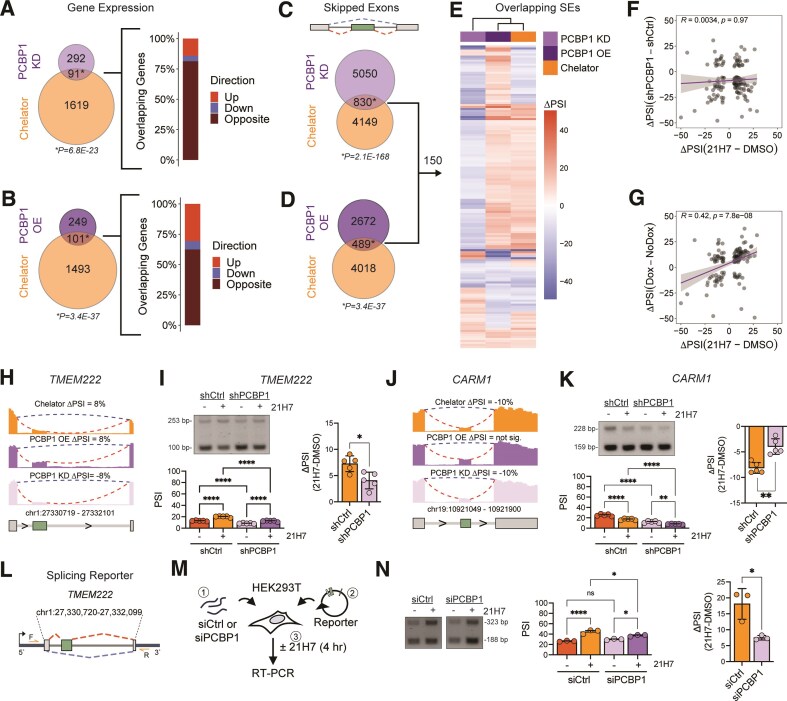
Iron-sensitive PCBP1-regulated splicing events. (**A**) Venn diagram of significantly DEGs in chelation and PCBP1 KD and bar plot of overlapping genes with direction of regulation (upregulated, red; downregulated, blue; or opposing regulation, brown). Hypergeometric test used to calculate overlap significance. (**B**) Venn diagram comparing significantly DEGs in chelation and PCBP1 OE and bar plot of overlapping genes with direction of regulation (upregulated, red; downregulated, blue; or opposing regulation, brown). Hypergeometric test used to calculate overlap significance. (**C**) Venn diagram comparing significant SEs in Chelator and PCBP1 KD. (**D**) Venn diagram comparing significant SEs in Chelator and PCBP1 OE. Hypergeometric test used to calculate overlap significance. (**E**) Heatmap with clustering of significant SEs between Chelator, PCBP1 KD, and PCBP1 OE. Red is exon inclusion and blue is exon exclusion. (**F**) Scatter plot of significant SEs between Chelator and PCBP1 KD from panel E with Spearman’s rank correlation. (**G**) Scatter plot of overlapping significant SEs between Chelator and PCBP1 OE from panel E with Spearman’s rank correlation. (**H**) RNAseq coverage tracks of *TMEM222* after iron chelation, PCBP1 OE and KD, respectively. Mean ΔPSI of each condition reported. (**I**) Left, RT-PCR analysis and quantification of *TMEM222* splicing with or without PCBP1 KD and with or without 21H7, *n* = 5. ****P<.0001 by one way ANOVA. **Right**, Quantification of *TMEM222* mean ΔPSI after iron chelation with or without PCBP1 KD, *n* = 5, *P<.05, by two-tailed *t*-test. (**J**) RNAseq coverage tracks of *CARM1* after iron chelation, PCBP1 OE and KD, respectively. Mean ΔPSI of each condition reported. (**K**) Left, RT-PCR analysis and quantification of *CARM1* with or without PCBP1 KD and with or without 21H7, *n* = 5. **P= 0.0064, ****P<.0001 by one way ANOVA. **Right**, Quantification of *CARM1* mean ΔPSI after iron chelation with or without PCBP1 KD, *n* = 5, **P<.002, by two-tailed *t*-test. (**L**) Schematic of the *TMEM222* splicing reporter. F and R reflect the positions of the primers used. (**M**) Schematic of the reporter experiment in HEK293T cells. (**N**) Left, RT-PCR analysis and (**middle**) quantification of splicing reporter with or without PCBP1 KD and with or without 21H7, *n* = 3, ns not significant, *P<.05, ****P<.0001 by one way ANOVA. **Right**, Mean ΔPSI, *P<.03 by two-tailed *t*-test.

To assess the iron sensitivity of PCBP-dependent mRNA regulation, we integrated changes in gene expression and AS between iron chelation and PCBP1 KD or OE. There was a significant overlap in differential gene expression between iron chelation and PCBP1 KD, and interestingly, most of these genes were upregulated or downregulated in opposing directions (Fig. 3A). We similarly found a significant overlap between DEGs with iron chelation and PCBP1 OE (Fig. 3B). While many genes were upregulated or downregulated in opposing directions, there was a greater proportion of genes changing in expression in the same direction. These data suggest that, compared to KD, PCBP1 OE better mimics gene expression changes with iron chelation raising the possibility that iron dampens PCBP1-mediated control of gene expression.

Next, we examined significantly SE events between iron chelation and PCBP1 KD or OE. We found a significant overlap in SE events between PCBP1 KD and iron chelation (830 exons; Fig. 3C). Similarly, we found a significant overlap in events between PCBP1 OE and iron chelation (489 exons, Fig. 3D). In overlapping SE events between iron chelation, PCBP1 KD, and OE, we found 150 SE events. We next evaluated the specific inclusion or exclusion (ΔPSI) of these 150 events in all three datasets and found that PCBP1 OE clustered best with iron chelation (Fig. 3E and Supplemental Table S7). Next, we examined the correlation of the ΔPSI of the 150 events between each pair of datasets. There was no significant correlation in SE usage between PCBP1 KD and iron chelation (Fig. 3F). Consistent with splicing between iron chelation and PCBP1 OE clustering together, there was a significant positive correlation in SE usage between PCBP1 OE and iron chelation (Fig. 3G). Thus, if PCBP1 OE promoted exon inclusion, so too did iron chelation. These splicing data mirror our observation of similarities in differential gene expression between PCBP1 OE and iron chelation and further support the idea that iron availability impacts PCBP1 mRNA processing.

Pulling from our list of 150 SE (Fig. 3E), we identified an alternative exon in *TMEM222*. This gene encodes a poorly understood transmembrane protein associated with neurodevelopmental disorders. In the iron chelation and PCBP1 OE RNAseq data, we saw this alternative exon with increased inclusion, whereas PCBP1 KD decreased inclusion (Fig. 3H). We then combined iron chelation with PCBP1 KD, to look for attenuation of iron-chelation dependent changes in exon inclusion with loss of PCBP1. For the *TMEM222* SE, inclusion was increased with iron chelation in shCtrl cells, but that increase was reduced with shPCBP1 (Fig. 3I). We tested another SE in *CARM1—*a histone arginine methylase. We found increased skipping with iron chelation and PCBP1 KD while PCBP1 OE caused no change in inclusion in the RNAseq data (Fig. 3J). However, when we combined iron chelation with PCBP1 KD, we found the chelation-induced exclusion was blunted by PCBP1 KD (Fig. 3K). In both cases, iron chelation-induced splicing was partially attenuated by loss of PCBP1.

We next built a mini-gene splicing reporter using the alternative *TMEM222* exon (Fig. 3L). We transfected this reporter into HEK293T cells after PCBP1 siRNA or nontargeting siRNA treatment, followed by a short iron chelation treatment (Fig. 3K). Inclusion of the reporter alternative exon was increased 18 ± 5% with iron chelation and, although we did not detect a decrease of inclusion with PCBP1 KD, we did detect significant blunting of the iron chelation-induced exon inclusion (Fig. 3N). In sum, these data show the contribution of PCBP1 in control of AS during iron chelation.

### Iron-sensitive RNA regulation by PCBP1 and PCBP2

PCBP1 and PCBP2 share a considerable degree of amino acid homology (83%), especially across their RNA binding domains (93%) [69]. Similarly, both proteins show iron chaperone activity [8]. Thus, to further evaluate the connection between iron chelation and the PCBP-directed transcriptome, we examined PCBP2 KD in K562 cells from the ENCODE consortium [21]. We detected 560 significantly DEGs (Supplemental Fig. S2A and Supplemental Table S8) involved in catabolic processes and metabolism (Supplemental Fig. S2B and Supplemental Table S3). GSEA found negative enrichment for heme metabolism (Supplemental Fig. S2C) and oxidative phosphorylation, among others (Supplemental Table S2). Compared to PCBP1, the loss of PCBP2 caused a notable increase in retained introns although many other splicing changes were detected (Supplemental Fig. S2D–F). When we integrated PCBP2 KD data with iron chelation data, we found a significant overlap in genes regulated at the mRNA level (Supplemental Fig. S2G). Among SE, we found a significant overlap between iron chelation and shPCBP2 (Supplemental Fig. S2H), further suggesting that PCBP-regulated exons are differentially spliced when iron levels are low. As above, we compared the inclusion or exclusion level for SE under iron chelation versus PCBP2 KD. For overlapping events, we found a propensity for opposing exon inclusion between iron chelation and PCBP2 KD (Supplemental Fig. S2I). For example, if an exon was more included with iron chelation, it was likely excluded with PCBP2 KD.

**Figure 4.**
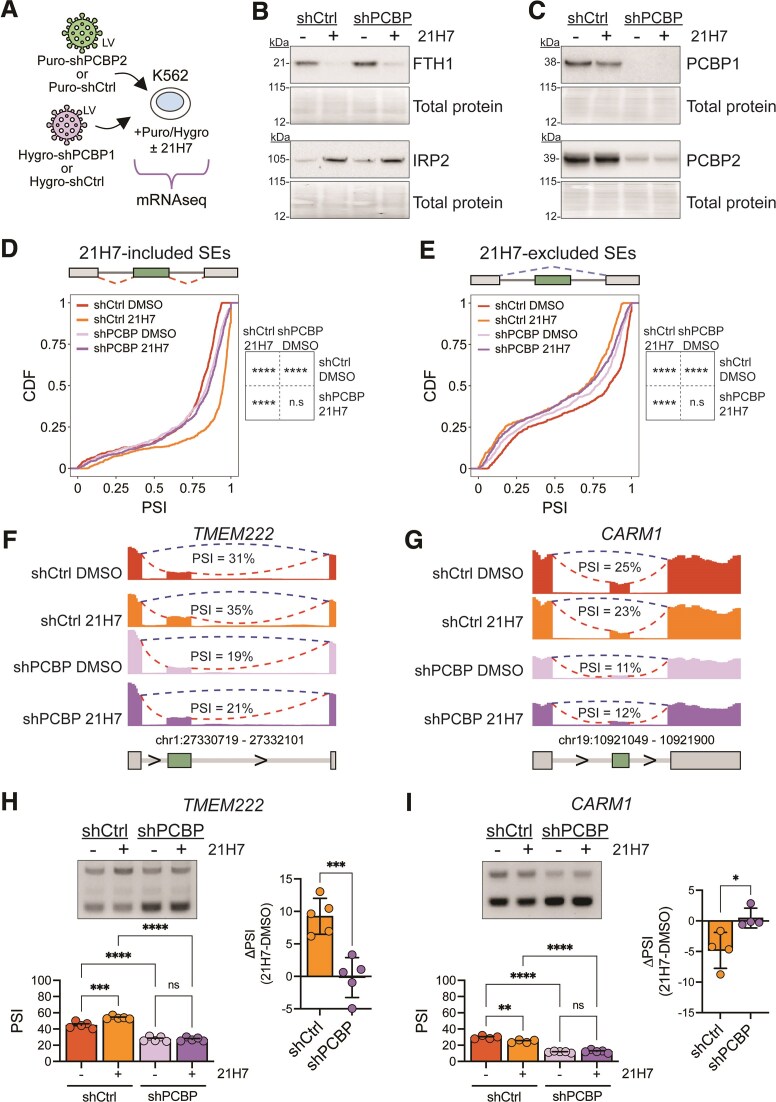
Iron-sensitive PCBP-regulated AS. (**A**) Schematic of the mRNA-seq experiment of K562 cells transduced with Puro-shPCBP2 and Hygro-shPCBP1 or Puro-shCtrl and Hygro-shCtrl, with or with iron chelation, *n* = 3. (**B**) Western blots of FTH1 and IRP2 after PCBP1 and PCBP2 KD or control with or without iron chelation. Total protein was used as a loading control. (**C**) Western blots of PCBP1 and PCBP2 after PCBP1 and PCBP2 KD or control with or without iron chelation. Total protein was used as a loading control. (**D) Left**, cumulative distribution function (CDF) of chelation-induced SE inclusion. **Right**, Kolmogorov–Smirnov (KS) test matrix. n.s. = not significant, ****P<.00001. (**E**) Left, CDF of chelation-induced SE exclusion. **Right**, KS test matrix. n.s. = not significant, ****P<.00001. (**F**) RNAseq coverage tracks of *TMEM222* with mean PSI reported. (**G**) RNAseq coverage tracks of *CARM1* with mean PSI reported. (**H**) Left, RT-PCR analysis and quantification of *TMEM222* with or without PCBP1 and PCBP2 KD and with or without 21H7, *n* = 5. ***P= 0.0004, ****P<.0001 by one-way ANOVA. **Right**, Quantification of *TMEM222* mean ΔPSI after iron chelation with or without PCBP1 and PCBP2 KD, *n* = 5, ***P= 0.0009 by two-tailed *t*-test. (**I**) Left, RT-PCR analysis and quantification of *CARM1* with or without PCBP1 and PCBP2 KD and with or without 21H7, *n* = 5. **P= 0.003, ****P<.0001 by one-way ANOVA. **Right**, Quantification of *CARM1* mean ΔPSI after iron chelation with or without PCBP1 and PCBP2 KD, *n* = 4, *P= 0.028 by two-tailed *t*-test.

Given the potential for iron-sensitive RNA regulation by both PCBPs, we performed deep mRNAseq after dual KD of PCBP1 and PCBP2 in K562 cells with or without 21H7. This was achieved using two sets of lentiviral particles expressing shPCBP1 or shPCBP2 (and their respective shCtrl particles) with different antibiotic resistance genes, *HygR* and *PuroR*, respectively (Fig. 4A, *n* = 3). Co-transduced cells were then selected with hygromycin and puromycin dual treatment. Four days later, co-transduced cells were treated with 10 μM 21H7 or DMSO overnight followed by RNA isolation and mRNAseq. We first confirmed effective 21H7 treatment by measuring FTH1 and IRP2 protein expression level changes (Fig. 4B and Supplemental Fig. S3A). In both shCtrl and shPCBP, we observed loss of FTH1 and increased IRP2 in response to iron chelation. We also confirmed effective KD of PCBP1 and PCBP2 protein levels (Fig. 4C and Supplemental Fig. S3B). Compared to nontransduced cells (Fig. 1D), we observed fewer DEGs after iron chelation in shCtrl cells (Supplemental Fig. S3C and Supplemental Table S9), however, as above, we observed more upregulated than downregulated genes (592 versus 285, respectively, *P <*2.2e-16 by Chi-squared test). Dual KD of PCBPs drove significant changes in gene expression (1214 genes up, 917 genes down, *P* = 1.24e-10 by Chi-squared test), especially compared to KD of either protein alone (Supplemental Fig. S3C versus Fig. 2C and Supplemental Fig. S2A), suggesting a level of compensation between these paralogs.

We next evaluated AS events across these four conditions (Supplemental Fig. S3D–G). We asked how many significant splicing events were chelation-sensitive in co-transduced shCtrl cells. Among SE, we detected 1425 events, with slightly more exons included with 21H7 treatment than skipped (723 included, 702 skipped, *P*= 0.578 by Chi-squared test), but overall fewer alternative exons were detected compared to nontransduced cells (Fig. 1K). We then compared the inclusion level of these chelation-sensitive SE across samples to evaluate if dual PCBP loss could attenuate iron chelation-induced effects. For chelation-induced SE inclusion, loss of PCBPs caused an overall inhibition of inclusion (Fig. 4D), whereas for chelation-induced SE exclusion, PCBP loss had a less clear impact likely because both PCBPs primarily promote exon inclusion (Fig. 4E). Read coverage tracks for the alternative exons in *TMEM222* and *CARM1* showed PCBP loss fully negated chelation-induced AS (Fig. 4F and G). Again, we validated both splicing events, noting that loss of both PCBPs resulted in complete loss of chelation-sensitive splicing compared to PCBP1 KD alone (Fig. 3I versus 4H and 3K versus 4I). Taken together, these data support the idea that intracellular iron levels alter the mRNA regulatory activity of PCBPs. Specifically, these data suggest that low iron levels promote PCBP mRNA regulation, whereas iron replete levels may reduce it, perhaps through altered RNA binding.

### Low iron availability modulates PCBP1 association with RNA in cells

**Figure 5.**
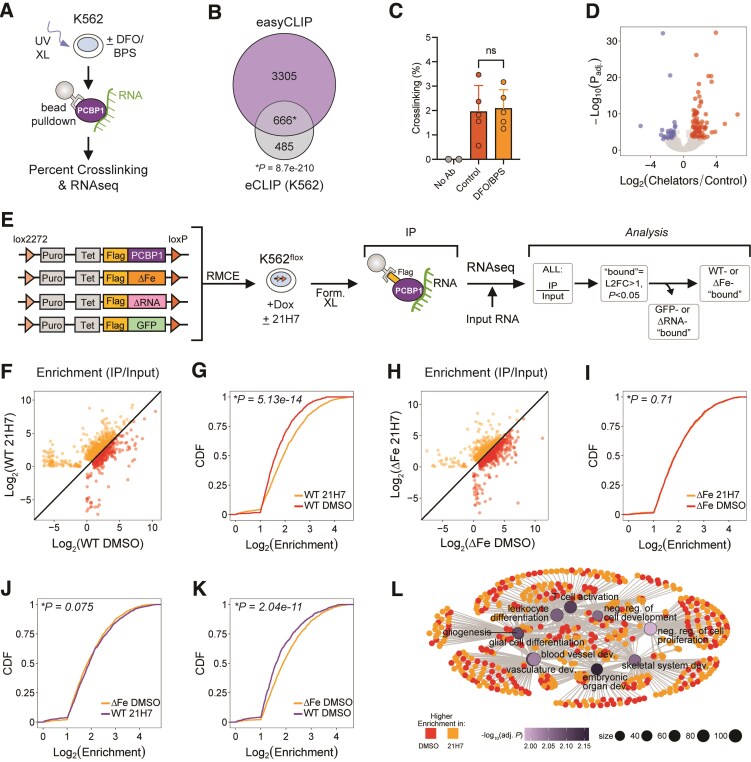
Iron chelation alters PCBP1 RNA association. (**A**) Schematic of the easyCLIP experiment. (**B**) Venn diagram of transcripts bound to PCBP1 by eCLIP (ENCODE) and transcripts with a DESeq2 basemean ≥5 in the easyCLIP. All detected transcripts with a DESeq2 basemean ≥5 were used as the universe for the hypergeometric test used for significance. (**C**) Percent of RNA crosslinking to PCBP1 in cells treated with or without iron chelators (DFO/BPS, *n* = 5). Data are mean ± SEM. n.s., not significant by two-tailed *t*-test. (**D**) Volcano plot of differentially bound genes to PCBP1 after control or DFO/BPS treatment, *n* = 3. Significance by Wald test with BH method for adjusted *P*-value. (**E**) Schematic of RNA-immunoprecipitation and sequencing (RIPseq) experiment using Flag-tagged WT, ΔFe, ΔRNA PCBP1, and GFP (*n* = 3), with analysis pipeline. (**F**) Scatter plot of genes significantly “bound” to WT PCBP1 with or without iron chelation. Orange represents genes better “bound” to WT PCBP1 in 21H7, and red represents genes better “bound” to WT PCBP1 in DMSO. Significance by Wald test with BH method for adjusted *P*-value. (**G**) CDF of genes significantly “bound” for WT PCBP1 with or without iron chelation. KS test used to determine significance. (**H**) Scatter plot of genes significantly “bound” to ΔFe PCBP1 with or without iron chelation. Orange represents genes better “bound” to ΔFe PCBP1 with 21H7, and red represents genes better “bound” to ΔFe PCBP1 in DMSO. Significance by Wald test with BH method for adjusted *P*-value. (**I**) CDF of genes significantly “bound” to ΔFe PCBP1 IP versus input with iron chelation versus without iron chelation. KS test used to determine significance. (**J**) CDF of genes significantly “bound” to ΔFe PCBP1 without iron chelation versus WT PCBP1 with iron chelation. KS test used to determine significance. (**K**) CDF of genes significantly “bound” to ΔFe PCBP1 without iron chelation versus WT PCBP1 without iron chelation. KS test used to determine significance. (**L**) Network plot of GO terms from transcripts significantly “bound” to WT PCBP1. GO term node size and color reflect transcript (at gene level) count and significance, respectively (over-representation test with adjusted *P*-value by BH method). Genes in each node colored if their enrichment was higher with 21H7 (orange) or DMSO (red) and gray lines connect each gene to their associated GO term. For CDF plots in panels (G), (I), (J), and (K), statistical outliers were removed for visualization purposes but were included in all statistical analyses.

To evaluate how RNA binding by PCBP1 is altered by low iron availability in cells, we turned to a quantitative UV crosslinking and IP method (easyCLIP [40]) (Fig. 5A). As with other crosslinking and IP approaches (such as eCLIP), easyCLIP involves UV crosslinking (which covalently links protein and RNA in direct interactions), IP of a target RBP and isolation of the bound RNA followed by library preparation. However, easyCLIP also incorporates a method to quantify how many moles of RNA are crosslinked per mole of protein and, therefore, to calculate a percent of crosslinking. We performed easyCLIP with K562 cells treated with or without iron chelators (DFO and BPS, *n* = 5). We sequenced the bound RNAs (*n* = 3) and validated our hits, at the gene level, against the ENCODE K562 PCBP1 eCLIP experiment. We found a significant overlap between easyCLIP targets and eCLIP targets (Fig. 5B). Next, we determined the percent of RNA crosslinking to PCBP1 in cells treated with or without iron chelation. Compared to control, there was no difference with iron chelation in overall RNA crosslinking to PCBP1 (Fig. 5C). However, with low iron there were 85 gene transcripts more bound to PCBP1, whereas 29 gene transcripts were less bound (Fig. 5D). These data suggest PCBP1 was more bound to at least a subset of target RNAs after iron chelation. However, of note, we did not determine if bound genes also changed in expression from these samples.

Because PCBP1 has a very low UV crosslinking rate compared to other RBPs [40], we turned to formaldehyde crosslinking followed by RIPseq. This approach captures both direct and indirect RNA-protein interactions. For this experiment, we used our Dox-inducible Flag-tagged wildtype (WT) PCBP1 cell line and similarly generated K562 cells with inducible expression of two additional PCBP1 variants, as shown in Supplemental Fig. S4A: (i) PCBP1 ΔFe with aspartate or glutamate mutations (D82A, E168A, E350A), one per KH domain, reported to reduce iron interactions [22], and (ii) PCBP1 ΔRNA with three arginine mutations (R40A, R124A, and R306A), one in each KH domain, that drastically reduces binding to C-rich RNA [22]. The ΔRNA cell line as well as a Dox-inducible Flag-GFP cell line served as background controls. All four cell lines were treated with Dox followed by DMSO or 21H7 treatment (*n* = 3), formaldehyde crosslinking, IP for the Flag-tagged proteins, and isolation of bound RNA (Fig. 5E). We first confirmed expression levels of Flag-tagged proteins and 21H7 treatment (Supplemental Fig. 4B–E). Flag-GFP expression was considerably lower than all other Flag-tagged proteins; however, for the IP step, we normalized cell lysates to Flag-GFP expression. To measure enrichments of PCBP1-bound transcripts, we compared the IP of each Flag-tagged protein to their respective inputs with and without chelation (Supplemental Fig. S4F–I). Transcripts with enrichments >1 (log_2_ fold change > 1, *P*_adj_ <.05) were considered “bound”. After filtering out GFP and ΔRNA-bound transcripts, we found 2830 gene transcripts were significantly associated with WT PCBP1 in DMSO treatment, 600 (21%) of which overlapped with eCLIP hits (Supplemental Fig. S4J), and 2766 gene transcripts associated with WT after iron chelation (Fig. 5F and Supplemental Table S10). Overall enrichments for WT-associated transcripts were increased under conditions of iron chelation (Fig. 5G). We further filtered our list of PCBP1-associated RNAs to focus on gene transcripts that did not change in expression in response to iron chelation. WT enrichments for these transcripts were significantly higher with iron chelation (Supplemental Fig. S4K). These data suggest low iron levels enhance PCBP1 association with RNA.

We next evaluated how ΔFe associates with RNA and found it bound 3269 gene transcripts with DMSO and 3243 gene transcripts with iron chelation (Fig. 5H and Supplemental Table S11). Similar to WT, 644 (20%) of ΔFe associated transcripts overlapped with eCLIP hits (Supplemental Fig. S4L). In contrast to WT, there was no difference in overall RNA enrichments for ΔFe with or without iron chelation (Fig. 5I). We reasoned that ΔFe, through loss of iron binding, should mimic WT with iron chelation; indeed, we found no difference in RNA enrichments between ΔFe DMSO and WT 21H7 (Fig. 5J and Supplemental Fig. S4M). Further, when we compared WT to ΔFe in control conditions, we found increased enrichments for ΔFe (Fig. 5K and Supplemental Fig. S4N). These data support a model in which iron interactions with PCBP1 reduces its association with RNA, however the specific molecular mechanism driving this is still to be determined.

A GO analysis on WT associated transcripts revealed several developmental pathways such as embryonic organ, skeletal system, and blood vessel development (Fig. 5L and Supplemental Table S12). For skeletal system development, 63 out of 99 gene transcripts had higher enrichment with WT PCBP1 after iron chelation (*P* = 0.006 by Chi-squared test). The numerous developmental pathway hits are consistent with PCBP1’s apparent role in embryogenesis—PCBP1 knockout causes peri-implantation lethality in mice [18]. PCBP1 ΔFe showed interactions with transcripts in pathways highly similar to WT (Supplemental Fig. S4O and Supplemental Table S13). Of note, we found important iron homeostasis transcripts among those associated with PCBP1, namely *FTH1, TFR2, HJV*, and *HAMP* (Supplemental Table S10). *TFR2, HJV* and *HAMP* all have critical roles in how the liver senses and regulates systemic iron levels (reviewed in [2]). These data further show how iron availability intersects with PCBP mRNA regulation. Further studies in more relevant cell types, e.g. hepatocytes, will be needed to ascertain how the potential for iron-sensitive PCBP interactions with these transcripts may contribute to iron homeostasis.

## Discussion

Iron deficiency is the most common nutritional deficit worldwide, commonly impacting children and women [70]. Compelling recent studies underscore how iron deficiency alters gene expression, particularly in the context of maternal iron deficiency. For example, murine embryos from iron-deficient dams exhibit transcriptomic changes in cardiac progenitor cells associated with cardiac malformation [71]. Another study showed how loss of iron-dependent histone demethylase activity in fetal gonads, caused by maternal iron deficiency, leads to ovary and mammary gland development in male neonatal mice [72]. It is highly likely there are other iron-sensitive developmental pathways yet to be described. Here we highlight the extensive RNA processing programs that are modulated by iron chelation and highlight PCBPs as candidate effectors.

Our work shows differential mRNA binding and AS by PCBP1 based on cellular iron availability. Yet, PCBPs have additional RNA regulatory functions, such as translational control of both cellular and viral mRNAs [19]. In particular, PCBP1 functions as a translational repressor [73, 74]. Notably, translation is globally repressed during iron deficiency [75]. Given our finding that reduced iron availability enhances PCBP1 RNA interactions, PCBPs could similarly contribute to translational repression during iron deficiency. Additionally, PCBPs regulate stability of mRNAs involved in a variety of cellular processes, including cytokine production and α-globin expression, among others [16, 76–78]. The potential for iron-sensitive regulation of mRNA stability through PCBPs may impact those same processes. Follow-up studies focused on translation and mRNA stability regulation will be needed to uncover whether these other PCBP functions are iron-sensitive.

We find it notable that PCBP1 and PCBP2 regulate the expression of genes involved in iron metabolism. While some of these gene expression changes could be interpreted as a response to altered iron levels due to loss of PCBPs as iron chaperones, some of the mRNAs involved in these pathways are directly bound by PCBP1 in this study and also identified in other studies [79, 80]. In addition, the PCBP-directed transcriptome involves transcription factor pathways (e.g. E2F), developmental pathways, catabolic processes and multiple iron or oxygen-related pathways (e.g. heme metabolism and hypoxia). PCBPs are therefore situated at the nexus of gene expression and metabolism. Thus, the role of PCBPs in regulating iron flux in the cell likely extends beyond their role as iron chaperones and may involve an iron-dependent mRNA regulon, akin to that of the IRPs [81].

Like other RBPs, PCBPs perform their RNA regulatory function as components of ribonucleoprotein complexes. For example, PCBP1 interacts with U2AF2 and the U2 snRNP at 3′ splice sites of alternative exons [45] and with PABP at 3′ untranslated regions to exert control of mRNA stability [82]. Although there is evidence of PCBP1 iron-sensitive protein-protein interactions [11], how the full PCBP interactome, particularly with RNA-related factors, responds to iron remains unknown. Further work is needed to elucidate how cellular iron regulates the PCBP protein interactome.

Although we think it probable that low iron levels can drive global changes in PCBP protein-protein interactions to favor enhanced RNA interactions, it is plausible that the iron-associated and RNA-associated pools of PCBP1 or PCBP2 act independently. As of now, the proportion of PCBP bound to RNA, iron, or free of either in the cell is unknown and likely to be dynamic. Due to a lack of biophysical and structural work on iron binding to PCBP1 it is difficult to draw specific conclusions on how ΔFe mutations in PCBP1 impact RNA binding. The localization of PCBP1 to both the nucleus and the cytoplasm and its known roles in iron homeostasis and RNA processing in both compartments further complicates understanding how these activities are intertwined.

Another possible mechanism for iron-sensitive RNA binding by PCBPs could be changes in post-translational modifications. For example, PCBP1 is phosphorylated at several sites and thus translocated into or out of the nucleus [74, 77]. The activity of some phosphatases is iron-dependent (e.g. protein phosphatase 1a [83]) suggesting the possibility that PCBP1 phosphorylation status may be sensitive to cellular iron levels. However, phosphatases that regulate PCBP1 phosphorylation have not yet been identified. Alternatively, the kinases or signaling pathways that drive PCBP phosphorylation may be altered after iron chelation.

Our study has several limitations. First, we used an iron chelator to lower available iron levels. Although commonly employed in this fashion, it is possible this approach does not fully capture the effects of iron deficiency and off target effects may have unexpected consequences. However, it is of note that iron chelators, like deferoxamine or deferiprone, are used clinically for the treatment of iron overload such as in transfusion-dependent patients with β-thalassemia. Thus, understanding the cellular response to iron chelation can have real world implications. In addition, we choose a 16-h duration for iron chelation; however, this timing may not capture early or transient events and may introduce secondary, nondirect effects. We did, however, find AS changes after 4 h of iron chelation. Another limitation is our use of a cancer cell line, K562, with known genomic anomalies [84] that likely influence the transcriptome and metabolism. However, our data from this cell line can be widely integrated with numerous other studies using K562 cells and used to generate hypotheses for testing in normal cells or tissues. An additional limitation is our use of polyA selection during library preparation for sequencing. PolyA selection may favor mRNAs with longer polyA tails, thus iron-sensitive splicing changes in mRNAs with shorter polyA tails may be underrepresented.

In summary, PCBPs are ubiquitously expressed RBPs that regulate the fate of many RNAs and are essential for life. Their roles as iron chaperones enable them to be highly functionally sensitive to iron levels. Our data suggest PCBPs integrate iron sensing into changes to the transcriptome through regulation of AS. Further work is needed to uncover the likely multifactorial mechanisms that confer this ability. Though PCBPs were the focus our study, the work we demonstrated can be used in the discovery of additional RBPs with iron-sensitive RNA regulation.

## Supplementary Material

gkaf942_Supplemental_Files

## Data Availability

RNAseq fastq files and processed data files are available at the NCBI Gene Expression Omnibus (GEO) under accession numbers GSE279949, GSE279951, GSE279953, GSE302393, and GSE302401. RNAseq analysis and plot scripts are available at https://doi.org/10.6084/m9.figshare.28143701.v1. Hypoxia RNAseq from Jain *et al.* [37] is available at GEO under accession number GSE144527. RNAseq after PCBP2 knockdown in K562 cells is available through ENCODE at https://doi.org/10.17989/ENCSR648QFY.

## Notes

Present address: University of Michigan, Ann Arbor, MI 48109, United States

Present address: Northwestern University, Chicago, IL 60208, United States
