# Supplementary material for: Iron-sensitive RNA regulation by poly C-binding proteins
Source: Nucleic Acids Res. 2025 Sep 30;53(18):gkaf942. doi: 10.1093/nar/gkaf942 (PMC12481019; doi:10.1093/nar/gkaf942)
Supplement: gkaf942_Supplemental_Files [file gkaf942_supplemental_files.zip › 250811_Supplemental_Figures.pdf]

Supplemental Figure S1

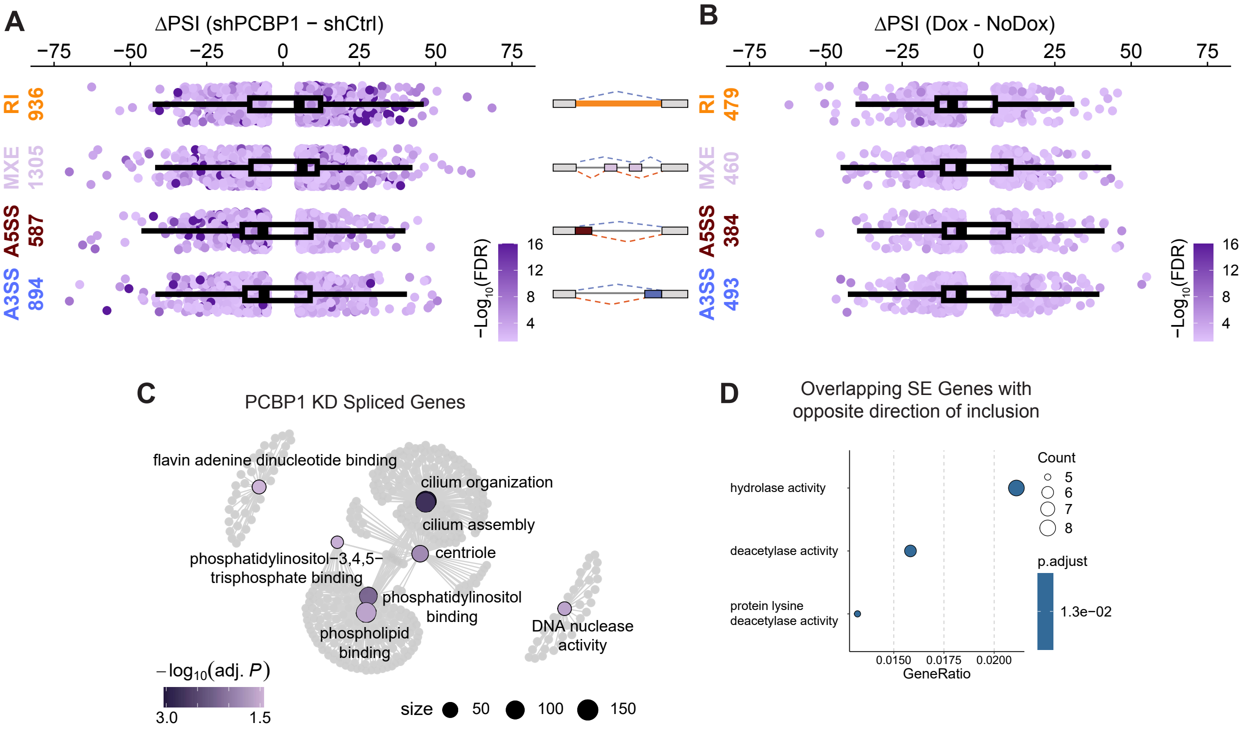

Supplemental Figure S1. Additional data for the PCBP1-regulated transcriptome. (**A**) Scatter and overlaid box plot of significant retained introns (RI), mutually exclusive exons (MXE), alternative 5′ splice sites (A5SS), and alternative 3′ splice sites (A3SS) events after PCBP1 knockdown. Each dot is an event colored by degree of significance (likelihood ratio test with FDR by Benjamin-Hochberg (BH) method). Box plot shows median of AS event PSI. (**B**) Scatter and overlaid box plot of significant RI, MXE, A5SS, and A3SS events after PCBP1 overexpression. Each dot is an event colored by degree of significance (likelihood ratio test with FDR by BH method). Box plot shows median of AS event PSI. (**C**) Network plot of GO terms from significant AS genes after PCBP1 knockdown GO term node size and color reflect gene count and significance (over-representation test with adjusted *P* value by BH method), respectively. Grey dots are individual genes, and grey lines connect each gene to their associated GO term. (**D**) Dot plot of GO terms from overlapping spliced exon genes (from PCBP1 knockdown and overexpression) with opposite direction of inclusion. Over-representation test with adjusted *P* value by BH method.

Supplemental Figure S2

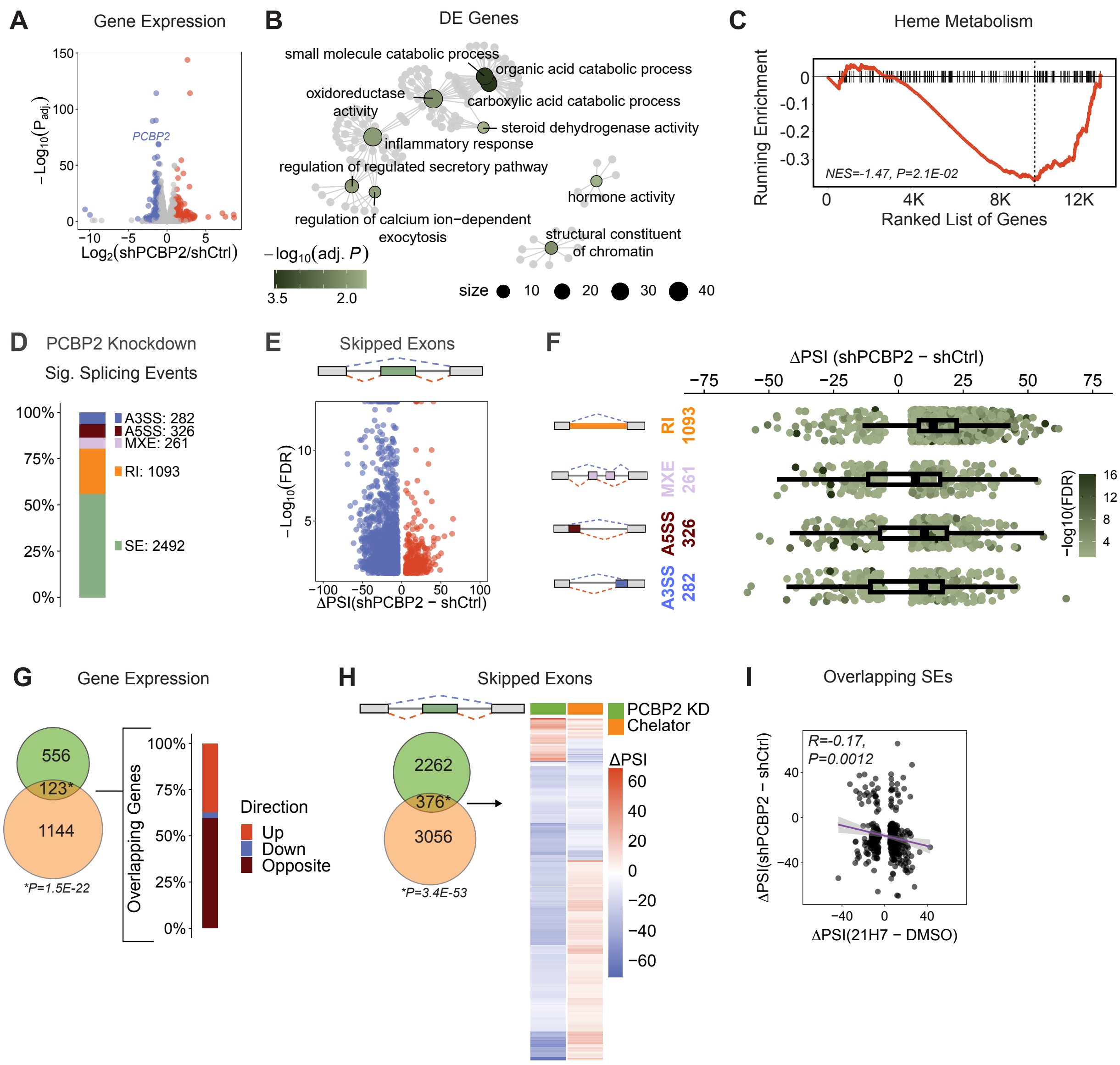

Supplemental Figure S2. The PCBP2-regulated transcriptome. (**A**) Volcano plot of DEGs after PCBP2 KD. Significance tested by Wald test with BH method for adjusted *P* values. (**B**) Network plot of GO terms from significantly DEGs after PCBP2 KD. GO term node size and color reflect gene count and significance, respectively (over-representation test with adjusted *P* value by BH method). Grey dots are individual genes, and grey lines connect each gene to their associated GO term. (**C**) Enrichment of 'hallmark' heme metabolism genes after PCBP2 KD. NES and adjusted *P* values calculated by GSEA. (**D**) Bar plot of the proportion and count of significant AS events with PCBP2 KD for each event type (by rMATS). (**E**) Volcano plot of significant SEs with PCBP2 KD. Significance by likelihood ratio test with FDR by BH method. (**F**) Scatter and overlaid box plots of significant RI, MXE, A5SS, and A3SS events after PCBP1 knockdown. Each dot is an event colored by degree of significance (likelihood ratio test with FDR by BH method). Box plot shows median of ΔPSI for each AS event. (**G**) Venn diagram of significantly DEGs in chelation and PCBP2 KD and bar plot of overlapping genes with direction of regulation (up-regulated, red; down-regulated, blue; or opposing regulation, brown). Hypergeometric test used to calculate overlap significance. (**H**) Venn diagram comparing significant SEs in Chelator and PCBP2 KD with heatmap of event ΔPSI. Red is exon inclusion and blue is exon exclusion. Hypergeometric test was used to calculate overlap significance. (**I**) Scatter plot of significant SEs overlapping between Chelator and PCBP2 KD from panel H, with Spearman's rank correlation.

Supplemental Figure S3

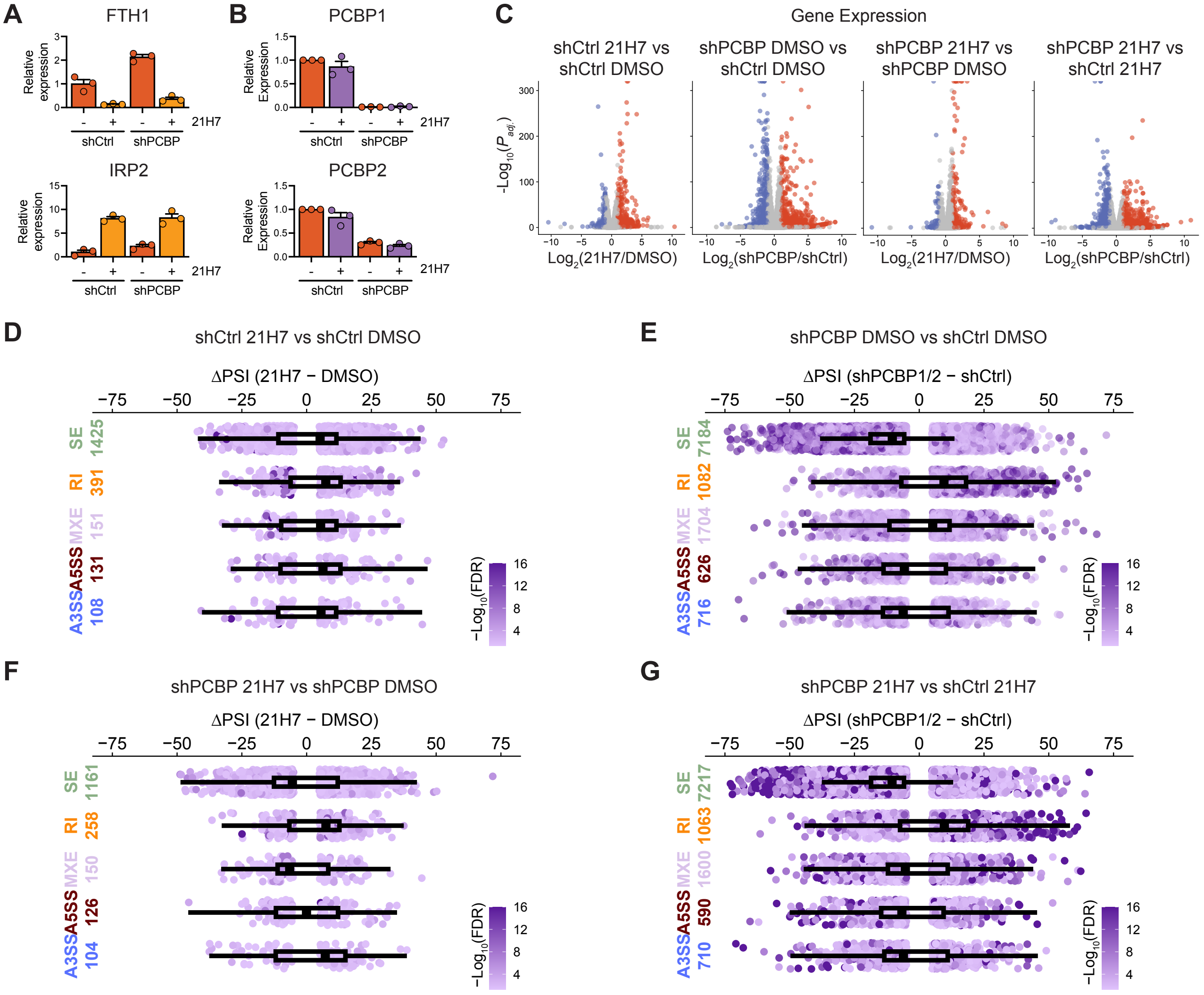

Supplemental Figure S3. Additional data for the iron-sensitive PCBP1/2-regulated transcriptome. (**A**) Quantification of FTH1 and IRP2 western blots shown in Figure 4B. (**B**) Quantification of PCBP1 and PCBP2 western blots shown in Figure 4C. (**C**) Volcano plots showing DEGs in each of the four conditions for the dual knockdown mRNAseq experiment. Significance tested by Wald test with BH method for adjusted *P* values. (**D-G**) Scatter and overlaid box plots of alternative splicing events in four different comparisons: D, effect of iron chelation in control cells; E, effect of dual knockdown; F, effect of chelation with loss of PCBPs; G, effect of knockdown with iron chelation. Each dot is an event colored by degree of significance (likelihood ratio test with FDR by BH method). Box plot shows median of ΔPSI for each AS event.

Supplemental Figure S4

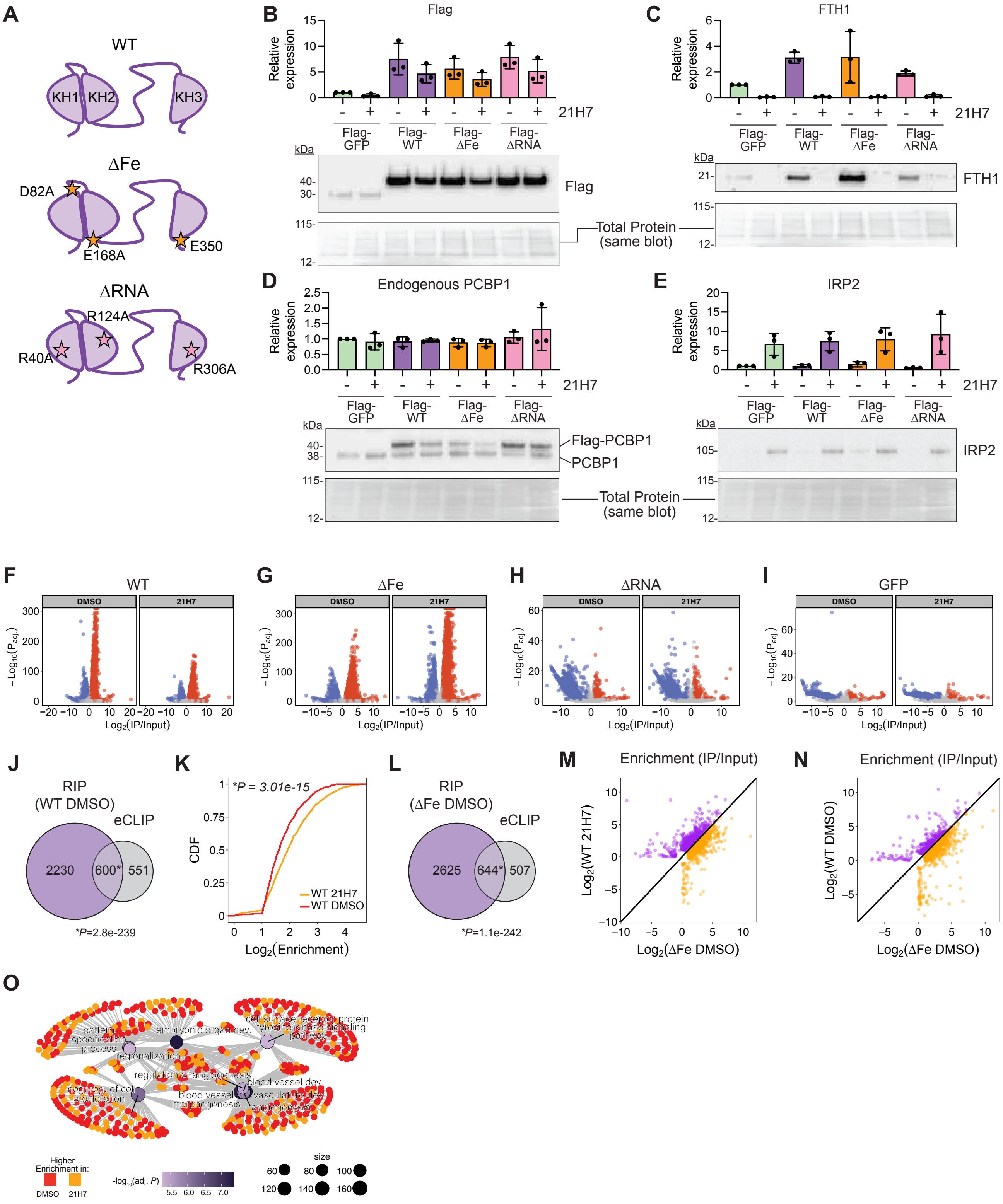

Supplemental Figure S4. Additional data on how iron chelation alters PCBP1 RNA association. (**A**) Schematics of tertiary structure of PCBP1 WT, ΔFe, and ΔRNA with associated mutations and their relative locations indicated. (**B-E**) Western blots of input cell lysates from RIPseq of Flag-tagged proteins (B), FTH1 (C), endogenous PCBP1 (D), and IRP2 (E). Blots used for total protein assessment by Ponceau S stain, were subsequently cut in two pieces for separate western blots for Flag and FTH1, or endogenous PCBP1 and IRP2, as indicated. (**F-I**) Volcano plots of “bound” transcripts (by gene) under DMSO or 21H7 conditions for WT (F), ΔFe (G), ΔRNA (H), and GFP (I). Red points are transcripts with positive enrichments while blue points are transcripts with negative enrichments. Significance tested by Wald test with BH

method for adjusted *P* values. (**J**) Venn diagram for WT DMSO “bound” transcripts in RIPseq and PCBP1 eCLIP. Hypergeometric test was used for significance, in which differentially expressed genes after iron chelation with a DESeq2 basemean >= 5 was used as the universe set of genes. (**K**) CDF of WT DMSO and WT 21H7 enrichments limited to genes that do not change in overall mRNA level. KS test was used for significance. Statistical outliers were removed for visualization purposes but were included for statistical analysis. (**L**) Venn diagram for ∆Fe DMSO “bound” genes in RIPseq and PCBP1 eCLIP Hypergeometric test was used for significance, in which differentially expressed genes after iron chelation with a DESeq2 basemean >= 5 was used as the universe set of genes. (**M**) Scatter plot of “bound” transcripts enrichments comparing ∆Fe DMSO versus WT 21H7. (**N**) Scatter plot of “bound” transcript enrichments comparing ∆Fe DMSO versus WT DMSO. (**O**) Network plot of gene ontology (GO) terms from transcripts significantly “bound” to ∆Fe PCBP1. GO term node size and color reflect transcript (at gene level) count and significance (adjusted *P* value by BH test), respectively. Genes in each node colored if their enrichment was higher with 21H7 (orange) or DMSO (red) and grey lines connect each gene to their associated GO term.
